# Inhomogeneous spatio-temporal epidemic-type aftershock sequence model incorporating seismicity-triggering slow slip events

**DOI:** 10.1038/s41598-025-30205-z

**Published:** 2025-11-29

**Authors:** Isaías Bañales, Tomoaki Nishikawa, Yoshihiro Ito, Vladimir Kostoglodov, Ekaterina Kazachkina, José Santiago

**Affiliations:** 1 Disaster Prevention Research Institute, Kyoto University, Gokasho, Uji, 611-0011 Japan; 2 Instituto de Geofísica, Universidad Nacional Autónoma de México, 04510 Mexico City, Mexico

**Keywords:** ETAS, Slow earthquakes, Expectation maximization, Aftershocks, Point processes, Geophysics, Seismology

## Abstract

Clarifying the relationship between regular earthquakes and slow fault slip is essential for understanding the mechanisms behind seismic activity. We hypothesize that the background seismic activity is partially triggered by interplate slow-slip events (SSEs). Consequently, we present an extension of the spatio-temporal epidemic-type aftershock sequence (ETAS) model, which incorporates background seismicity as a piecewise constant function over time based on recent advances in the inference of space–time inhomogeneous point processes. In this study, Global Navigation Satellite System (GNSS) data is employed to identify the occurrence periods of SSEs, thereby delineating the intervals during which changes in background seismicity may occur. Due to the technical complexity of performing inference with an inhomogeneous ETAS model, this work employs a maximum likelihood inference method using the Expectation-Maximization (EM) algorithm. This approach also enables the inference of the branching process for aftershocks, allowing for the estimation of earthquake genealogy. This study elucidates how the background seismicity increases during the periods of SSEs in Guerrero, Mexico and Boso Peninsula, Japan, which allows for a more comprehensive understanding of seismic activity and the relationship between slow and fast earthquakes.

## Introduction

At the boundaries between tectonic plates, two types of spontaneous and episodic fault slip phenomena occur: fast (regular) earthquakes and slow earthquakes. These fault slip phenomena are closely related. Among slow earthquakes, those with a relatively large magnitude (approximately Mw 5 or greater) that can be detected geodetically are referred to as slow slip events (SSEs)^1^. It has been observed that SSEs often trigger small to moderate earthquakes in the Sagami Trough subduction zone in Japan^2^. Furthermore, SSEs have preceded and possibly triggered megathrust earthquakes at several subduction plate boundaries^3,4^.

Global Navigation Satellite System (GNSS) networks provide detailed daily information on crustal deformation and allow to detect and describe SSEs^5–8^. SSEs have also been discovered and studied in various regions worldwide, including, Middle America Trench^9^, the Japan Trench^10,11^, the Hikurangi subduction zone in New Zealand^12^ , and Peru^13^. In these regions, SSEs occurring at the plate boundary are thought to significantly impact seismic activity, and therefore quantifying the impact of SSEs is essential for improving the accuracy of earthquake forecasts.

The modeling and forecasting of fast-earthquake activity in a stochastic context has been widely accepted by the seismological community since the presentation of the seminal article by Ogata^14^, in which the epidemic-type aftershock sequence (ETAS) model is defined using the Hawkes process. Moreover, Zhuang et al.^15^ made an important extension to the ETAS model by the inclusion of a spatio-temporal component in the intensity function (i.e., seismicity rate). In addition, Li et al.^16^ have studied in detail the inference of the background intensity function (i.e., the background seismicity rate in seismological terms) in spatio-temporally inhomogeneous point processes, this work was a pillar in the development of the model presented in “Methodology” section.

The detection of aseismic transients and their relationship to seismicity have been extensively studied. There have been several studies of seismicity changes due to aseismic transients considering only the time domain^17–21^. For example, Okutani & Ide^22^ investigated the impacts of SSEs on seismic activity using the temporal ETAS model. They proposed a model called the boxcar model, in which the background seismicity rate increases in a boxcar-like manner during the slow slip period estimated from geodetic observations. Their approach is similar to that one presented by Mattews & Reasenberg^23^, who investigated the quiescence of microearthquakes through a temporally inhomogeneous Poisson process, using a piecewise constant function.

Nishikawa & Nishimura^24^ presented a variant of the ETAS model that explicitly links an increase in background seismicity to detected SSE. Although their research has made significant advances in the modeling and forecasting of seismic activity associated with SSEs, it does not account for the spatio-temporal changes that may occur in background seismic activity during SSE periods.

As described above, extensive research has been conducted on time-domain analysis. In this work, however, the focus is on spatio-temporal seismicity modeling.

Llenos & McGuire^25^ proposed a complex model that combined the ETAS model and the rate- and state-dependent friction seismicity model^26^ to detect seismicity rate changes induced by aseismic transients. They adopted a tricky approach to subtract the coseismically triggered seismicity rate estimated by the conventional spatio-temporal ETAS model from the total seismicity rate and related the residual seismicity rate to the rate- and state-dependent friction seismicity model.

It is important to highlight the contributions of Marsan et al.^27^ and Reverso et al.^28^ to the spatio-temporal modeling of seismicity associated with aseismic transients. Both papers propose a way to model the evolution of the seismicity using a mesh over space and time. They used the conventional spatio-temporal ETAS model throughout the entire period as a null model, and for each earthquake occurring at time $t_i$ in the location $(x_i,y_i)$, they fit a locally elevated background intensity using earthquake records where $(t_j,x_j,y_j)$ satisfy

$$\begin{aligned} |t_j-t_i|&<\frac{\tau }{2},\\ |x_j-x_i|&<\frac{\mathcal {L}}{2},\\ |y_j-y_i|&<\frac{\mathcal {L}}{2}, \end{aligned}$$

for all *n*, where $\tau$ and $\mathcal {L}$ are parameters that control the size of the spatio-temporal window. If the locally estimated background intensity significantly differs from that of the null model, as determined by the criterion specified in each study, they replace the background intensity of the null model by the locally estimated value within the vicinity. Additionally, Reverso et al.^29^ have presented a pioneering work relating the ETAS model with SSE, following the ideas presented in^28^.

Possible improvements to the methods of Marsan et al.^27^ and Reverso et al.^28^ include the utilization of geodetic observations. In their methods, $\tau$ and $\mathcal {L}$ are subjectively chosen (e.g., $\tau$ is set to 1 day, 40 days, or 100 days). However, particularly for $\tau$, the duration of an aseismic transient can sometimes be estimated based on geodetic data, which can then be used as $\tau$.

Furthermore, they use the small spatio-temporal windows for each earthquake one by one to estimate the local background intensity via maximum likelihood estimation, sequentially comparing it to the null model. However, this is an approach adopted for simplicity. Ideally, the background intensities of multiple windows should be varied simultaneously to estimate the set of background intensities that maximize the likelihood.

In light of the aforementioned studies, this research proposes a new modification of the spatio-temporal ETAS model that incorporates the impact of SSEs on seismic activity (“Model” section). Our model determines the periods in which the background intensity changes based on GNSS observations and simultaneously estimates the spatial distribution of the background seismicity rate for each period. This model is mathematically grounded by Li et al.^16^.

We apply the new model to Mexican earthquakes in the Middle America subduction zone and elucidate the impact of SSEs on the background seismicity within this subduction zone, which is a topic that has not been addressed from the perspective of statistical modeling to date. In addition, the model was applied to the Boso Peninsula, located in the Sagami Trough subduction zone in Japan, a region that has been previously studied and has exhibited substantial changes in the seismic activity during SSE^22,30^. Our new model will be a useful tool in the future for elucidating the characteristics of seismic activity associated with SSEs worldwide.

## Methodology

### Introduction to spatio-temporal ETAS Model

The spatio-temporal ETAS model is a marked branching point process for earthquake occurrences, and its behavior can be completely defined through its conditional intensity function given by

1 $$\begin{aligned}&\mathbb {P}(\text {an event in }[t,t+dt]\times [x,x+dx]\times [y,y+dy]\nonumber \\&\quad \times [M,M+dM]|\mathcal {H}_t)=\lambda (t,x,y,M|\mathcal {H}_t)dtdxdydM+o(dtdxdydM), \end{aligned}$$

where *M* is the magnitude, (*x*, *y*) denotes the spatial coordinates, *t* represents the elapsed time, and $\mathcal {H}_t$ denotes the space-time and magnitude occurrence history of the earthquakes up to time *t*^15^. In particular, based on its assumptions, the spatio-temporal ETAS model has

2 $$\begin{aligned} \lambda (t,x,y|\mathcal {H}_t)=\mu (x,y)+\sum _{\{k:t_k<t\}} k(M_k)g(t-t_k)f(x-x_k,y-y_k|M_k), \end{aligned}$$

where the subindex *k* refers to the values of the $k-$th event considered, with $k=1,2,...,n$, $(x_k,y_k)$ are its spatial coordinates, $t_k$ is the time of occurrence, and $M_k$ is its magnitude. Without loss of generality, in this work it is assumed that $t_k$, $k=1,2,...,n$ are ordered in increasing order. Furthermore,

3 $$\begin{aligned} k(M)&=Ae^{\alpha (M-M_0)} \end{aligned}$$

4 $$\begin{aligned} g(t)&=(p-1)c^{p-1}(t+c)^{-p}\mathbbm {1}(t>0)\end{aligned}$$

5 $$\begin{aligned} f(x,y|M)&=\frac{1}{2\pi \sqrt{d_1d_2}e^{\alpha (M-M_0)}}\exp \Big \{-\frac{1}{2}\frac{1}{e^{\alpha (M-M_0)} }\Big (\frac{x^2}{d_1}+\frac{y^2}{d_2}\Big ) \Big \}, \end{aligned}$$

where $M_0$ denotes a reference magnitude, typically the minimum observed value, although in some cases a higher cutoff magnitude is adopted to reduce computational load^31^, and

$$\begin{aligned} \mathbbm {1}(t>0)={\left\{ \begin{array}{ll} 1, & \text {if } t>0\\ 0, & \text {otherwise} \end{array}\right. }. \end{aligned}$$

Different authors^15,32^ have used the Gaussian probability density function in equation (5). In this work, we assume that $d_1=d_2$ for the sake of parsimony, since the aftershock classification does not show significant differences compared to the case $d_1 \ne d_2$ for the Mexican data, as can be seen in Figs. 6 and 14a.

Another popular option is to use a Pareto distribution^33–35^, given by

$$\begin{aligned} f_{\text {Pareto}}(x,y|M)=\frac{q-1}{\pi \sqrt{d_1d_2} e^{\gamma (M-M_0)}}\Big (1+\frac{1}{e^{\gamma (M-M_0)}}\Big (\frac{x^2}{d_1}+\frac{y^2}{d_2}\Big )\Big )^{-q}, \end{aligned}$$

which can also be seen as a particular case of the bivariate t-distribution. The advantage of using the Pareto distribution is to avoid overestimate the background seismicity function. Nevertheless, for our Mexican data, the heavy tails of the Pareto distrubtion classify as aftershocks earthquakes that are unrealistically far from their respective mainshocks as it can be seen in Fig. 14b in Appendix App. 1.

By defining $\mu (x,y)=\nu u(x,y)$ and assuming stationarity, Zhuang et al.^15^ propose the estimator of $\mu$ as

6 $$\begin{aligned} \hat{\mu }(x,y)=\frac{1}{T} \sum _j (1-\rho _j) \frac{1}{2\pi d_j^2}\exp \Big \{-\frac{x^2+y^2}{2 d_j^2} \Big \}. \end{aligned}$$

where $d_j$ is a bandwidth that depends on how many earthquakes are close to the event *j*, and 1$-\rho _j$ is the probability that the $j-$event is an immigrant (i.e., background event).

$\hat{\mu }$ is fitted in an iterative two-step procedure, in the first iteration the vector $\eta =(\nu ,A,\alpha ,c,p,d)$ is fitted, in the second step the vector $\eta$ is taken as known and $\hat{\mu }$ is updated until the convergence of the log-likelihood

7 $$\begin{aligned} \ell (\eta ):=\sum _{k=1}^n \log (\lambda _{\eta }(t_k,x_k,y_k|H_{t_k}))-\int _0^T \iint _S \lambda _\eta (t,x,y|H_t)dxdydt, \end{aligned}$$

is reached, where the analysis time is [0, *T*], and *S* is the analysis region.

It is worth mentioning the novel nonparametric Bayesian approaches to model $\mu$. Ross & Kolev^32^ also assume that $\mu$ fulfills $\mu (x,y)=\nu u(x,y)$, where *u* is a probability density function and $\nu$ is a positive real number. This allows the use of a mixture of Dirichlet processes (MDP)^36^ as the prior for *u*(*x*, *y*). On the other hand, Molkenthin et al.^35^ assume that $\mu$ can be written as

$$\begin{aligned} \mu =\frac{v}{1+e^{-w(x,y)}}, \end{aligned}$$

where a Gaussian Process (GP) prior is used for *w*. The main advantage of using the MDP approach is that the function *u* always integrates 1, since it is a probability density function. In the case of GP, the integral can not be solved analytically; nevertheless, using the GP approach avoids the need for a finite approximation of the infinite mixture required in the MDP case.

It is important to mention that Veen & Schoenberg^37^ discuss in detail the numerical stability problems of maximizing the likelihood of the spatio-temporal ETAS model directly. They proposed using the Expectation-Maximization (EM) algorithm to improve the inference performance, this idea was one of our motivations to develop our model (“Model” section).

Extending the ideas presented by Veen & Schoenberg^37^, Fox et al.^38^ modeled the background intensity rate as a piecewise constant function, which allows a numerically efficient way to realize the approach proposed by Veen & Schoenberg^37^ with spatial inhomogeneities. The modeling of the background seismicity as a piecewise function is also used by different authors^39,40^ to infer the intensity function. Our approach (“Model” section) intends to advance the method proposed by Fox et al.^38^ by considering temporal inhomogeneities that will act as triggering effects due to SSEs.

Another advantage in following the approach presented by Veen & Schoenberg^37^ and Fox et al.^38^ is that the structure of the branching process is inferred in addition to estimating the intensity function. Therefore, we can easily distinguish which earthquakes are background events and which are aftershocks, as shown in the results obtained in “Results” section.

Since the ETAS model was introduced by^14^, the completeness of catalogs remains crucial for the correct estimation of the model. This is the reason that authors such as Seif et al.^33^ have made significant efforts to analyze the consequences of missing recorded aftershocks on the biases of the estimators. In their work, they argue that a possible solution to avoid incompleteness in aftershock sequences after a strong earthquake is to use a large cutoff magnitude. However, this can result in some seismic activity as foreshocks are not being observed and it is important to consider that the inclusion of small earthquake reduce the bias in the ETAS model estimators.

Short-term aftershock incompleteness has been extensively studied in sismology^41,42^.This is a problem of blindness, whereby small earthquakes are undetectable when the signal is saturated by a strong earthquake. Empirical relationships have been propose to model the STAI effect^43^. In the context of the ETAS algorithm, in recent years Hainzl^31^ has proposed the ETASI model, which incorporates the STAI phenomena into the ETAS model by adding a temporary dependence on the number of expected events. The ETASI model has been recently extended by Asayesh et al.^44^, where the spatial kernel of the ETASI model was modified to incorporate information from the stress scalars.

Although STAI is an important phenomenon to take into account, the cutoff magnitudes used in this study are not low enough to appreciate this phenomenon as it can be seen in Fig. 12 in Appendix App. 1. Therefore, we chose to keep b-values and cutoff magnitudes constant throughout the entire period.

We also wish to point out that, in order to model spatial variation in aftershock activity parameters, authors such as Ogata^45^ and Ueda et al.^46^ extended the spatio-temporal ETAS model^15^, allowing parameters such as *p* and the productivity *K* to become spatially varying functions. However, the issue of identifiability in their approach required the imposition of a smoothness penalization on the functions *p*, and *K*. Nevertheless, extending $\mu$, *K*, and *p* to functions of (*x*, *y*, *t*) leads to identifiability problems, since changes in seismicity induced by strong earthquakes can occur abruptly in both space and time, rendering smoothness penalization ineffective. For this reason, our work only considers temporal inhomogeneity through $\mu$.

### Model

Following the idea of Nishikawa and Nishimura^24^, we model the inhomogeneities in the ETAS model through changes in the background seismic activity. The reason for this is that $\mu$ is usually related to tectonic loading and the relative velocity of plate motion^47^. If we regard slow slip as an increase in tectonic loading or interplate slip rate, it is natural to model it through changes in the background seismic activity. However, we cannot deny the impact of SSEs on aftershock activity. Addressing this issue is an important direction for future work.

Since the aim of this work is to model the triggering effect of SSEs, the assumption of stationarity may not be realistic. In addition, Veen & Schoenberg^37^ have discussed that the approach introduced by Zhuang et al.^15^ is numerically expensive and unstable because it is necessary to optimize (7).

To describe the triggering effect due to SSEs, this work proposes to use

$$\begin{aligned} \lambda (t,x,y|\mathcal {H}_t)=\mu (x,y,t)+\sum _{\{k:t_k<t\}} k(M_k)g(t-t_k)f(x-x_k,y-y_k|M_k), \end{aligned}$$

which is an extension of (2) because it allows a time dependent $\mu$ function. However, due to the complexity of working with an arbitrary form of $\mu$, in this study, it is defined as

8 $$\begin{aligned} \mu (x,y,t)= \sum _{i=0}^m \mu ^i(x,y)\mathbbm {1}(t\in S_i,E_i), \end{aligned}$$

where *m* is the total number of SSEs and $S_i,E_i$ are the start and end times of the $i-$th SSE, with $i=1,...,m$. In the case of $i=0$, $S_i=0,E_i=T$, i.e. $\mu ^0$ represents the background seismicity over the entire period, regardless of the presence of an SSE, and the remaining $\mu ^i$ with *i*=1,2,..., *m* represent the increase of the background seismicity with respect to $\mu ^0$. Thus, the intensity is given by

9 $$\begin{aligned} \lambda (t,x,y|\mathcal {H}_t)=\sum _{i=0}^m \mu ^i(x,y)\mathbbm {1}(t\in S_i,E_i) +\sum _{\{k:t_k<t\}} k(M_k)g(t-t_k)f(x-x_k,y-y_k|M_k). \end{aligned}$$

As presented by Veen & Schoenberg^37^ and discussed in detail by others^48,49^, the Hawkes process could be defined as a marked Poisson cluster process where there are two kind of events, immigrants (background events) and offspring (aftershocks), which allows the use of the EM algorithm to maximize the likelihood.

McLachlan & Krishnan^50^ discussed the EM algorithm in detail and showed that it is useful when there is missing information which, if known, makes the maximization of the complete likelihood easier. The EM algorithm is an iterative algorithm that maximizes the likelihood using a two-step procedure, the first step (Expectation) replaces the unknown information by the expected one with the help of an initial value for all the parameters, and the second step (Maximization) uses the expected values obtained in the previous step to optimize the likelihood defined by the augmented information, these two steps are repeated until the convergence of the augmented likelihood.

The development of the model in this section is mathematically based on that proposed by Li et al.^16^, but the definition of $\lambda (t,x,y|\mathcal {H}_t)$ by them differs from that presented in (9). In their work they assumed

$$\begin{aligned} \mu (x,y,t)=\alpha u(x,y)v(t), \end{aligned}$$

where $\alpha$ is in $\mathbb {R}^+$, and *u* and *v* are positive functions. Since SSEs are spontaneous large fault slip events with variable slip evolution, the spatial and temporal contributions to background seismicity cannot be assumed to form a product over the entire period. Therefore, we preferred to use the expression in (8).

It is also important to mention that they are not working with a marked point process and that their intensity function does not incorporate information about event magnitudes. However, they use the kernel of a gamma distribution for the decay in the aftershock activity over the time with parameter $\alpha =1$:

$$\begin{aligned} g^L(t)=\beta e^{-\beta t} \end{aligned}$$

where $\beta$ is in $\mathbb {R}^+$. Their expression differs from (4), proposed by Zhuang et al.^15^, which is based on Omori’s law^51^ and is also adopted in our study.

From the observed data, it is not known whether an earthquake is a background event or an aftershock, and if it is a background event, it is also not known whether it comes from the process with intensity $\mu ^0$ or from $\mu ^i$ when the $i-$th SSE occurs. Therefore, the following random variables are defined:

10 $$\begin{aligned} \chi ^s_{ii}&={\left\{ \begin{array}{ll} 1, & \text {if earthquake }i\text { is a background event, and it is produced by } \mu ^s\\ 0, & \text {otherwise} \end{array}\right. }\end{aligned}$$

11 $$\begin{aligned} \chi _{ij}&={\left\{ \begin{array}{ll} 1, & \text {if earthquake }i\text { is an aftershock of }j\\ 0, & \text {otherwise} \end{array}\right. }, \end{aligned}$$

where $$s=0,...,m$$.

**Fig. 1:**
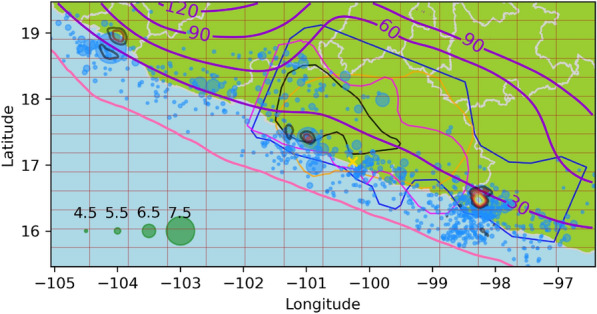
Seismicity data from Guerrero, Mexico. Earthquakes epicenters^67^ are shown in blue. Green circles are magnitude scale. The red squares define the grid used in Algorithm 1. The pink line denotes the Middle America Trench^71^. The gray lines show the political division of Mexico^72^, the blue, orange, fuchsia contours are the 4 cm slip contours curves digitalized from^73^ of the 2001-2002, 2006 and 2009-2010 SSE respectively, the black curve is the 15 cm slip contour of the 2014 SSE digitalized from^4^. The yellow cross correspond to the location of Cayaco GNSS station. The contour lines in purple are the slab model from^74^. We also present the slip distribution contours at 1-meter intervals from 1 to 6 meters for the earthquakes with $M>7$, using data from^67^. These earthquakes occurred on 2003/01/22, 2012/03/20, and 2014/04/18, with epicenters $-104.1040\hbox {E}^{\circ }$ $18.770\hbox {N}^{\circ }$, $-98.2310\hbox {E}^{\circ }$ $16.493\hbox {N}^{\circ }$, and $-100.9723\hbox {E}^{\circ }$ $17.397\hbox {N}^{\circ }$ and magnitudes 7.6, 7.4 and 7.2, respectively.

The random variables defined in (10) and (11) are an extension of those presented in the supplementary material by Fox et al.^38^, where only one $\chi _{ii}$ is introduced for the background intensity over the entire period, while here the triggering effect of multiple SSEs is modeled in a similar way to the model by Li et al.^16^. In the present work, $\mu ^i(x,y)$ is defined as a piecewise constant function for all *i*:

12 $$\begin{aligned} \mu ^i(x,y)=\sum _{u=1}^{n_y} \sum _{v=1}^{n_x} \mu _{uv}^i\mathbbm {1}((x,y)\in D_{uv}), \end{aligned}$$

where $D_{kl}=((u-1)\Delta x, u \Delta x)\times ((v-1)\Delta y, v \Delta y)$, $\Delta x$ and $\Delta y$ are the step size in the partition of the *x* and *y* axes, also $n_x$ and $n_y$ are the number of grids for each axis. The advantages of defining $\mu ^i(x,y)$ in this way is to facilitate the integral over the space in (17) to recover a closed expression in the maximization step, as it can be seen in (19). The grid applied in this study is shown in Fig. 1.

The total amount of parameters to be estimated by (12) are $n_y n_x$ for each $\mu ^i$, with $i\in 0,1,...m$. Furthermore, because of (3) to (5), we have the five aftershock parameters $(A,\alpha ,c,p,d)$ to be estimated. Additionally, we must estimate the whole branch structure given by $n(n-1)/2$ elements in $\chi _{ij}$ and $\sum _{i=1}^m n_i$ elements in $\chi ^s_{ii}$, where $n_i$ is the total number of earthquakes that occurred during $(S_i,E_i)$.

In this work, the array that contains all the parameters of the previous paragraph is denoted by $\theta$, i.e.

$$\begin{aligned} \theta =( \{\chi _{ii}\}_{i\in I},\{\chi _{ij}\}_{ij\in I\times I},A,\alpha ,c,p,d ). \end{aligned}$$

where $I=\{1,2,...,n\}$ and $\times$ denotes the cartesian product. It is important to note that the enormous amount of parameters will produce a not well-posed problem, the optimization of the likelihood has been constrained, following^52^ according to the expression (20). This study assumes that the parameters controlling the aftershock intensity $(A,\alpha ,c,p,d)$ do not change over time.

If the branch structure were known, the log-likelihood of the complete information ($\ell _c$) would be defined as

$$\begin{aligned} \ell _c(\theta )=\ell ^*_\text {O}(\theta )+\ell ^*_\text {I}(\theta ), \end{aligned}$$

where $\ell _\text {O}(\theta )$ is the log-likelihood due to the offspring, and $\ell _\text {I}(\theta )$ is the log-likelihood due to immigrants. They are given by

13 $$\begin{aligned} \ell ^*_\text {I}(\theta )&= \sum _{s=0}^m \Big [ \sum _{i=1}^n \chi _{ii}^s\log (\mu _\theta ^s(x_i,y_i)\mathbbm {1}(t_i\in (S_s,E_s))\nonumber \\&\quad -\int _0^T \iint _S \mu _\theta ^s(x_i,y_i)\mathbbm {1}(t\in (S_s,E_s)) dxdydt \Big ] \end{aligned}$$

14 $$\begin{aligned} \ell ^*_\text {O}(\theta )&= \sum _{j=1}^n \Big [ \sum _{i>j} \chi _{ij} \log \Big (k_{\theta }(M_j)g_{\theta }(t_i-t_j)f_{\theta }(x_i-x_j,y_i-y_j|M_j)\Big ) \nonumber \\&\quad - \int _{t_j}^T \iint _S k_{\theta }(M_j)g_{\theta }(t-t_j)f_{\theta }(x-x_j,y-y_j|M_j) dxdydt \Big ], \end{aligned}$$

where the subindex $\theta$ in $k_{\theta },g_{\theta },f_{\theta }$ and $\mu _\theta ^s$ means that the functions *k*, *g* and *f* are defined using the parameters $(A,\alpha ,c,p,d)$ given by the array $\theta$ where the log-likelihood is evaluated.

It is important to note that (10) and (11) are not independent random variables, the sum of all entries in the vector $(\chi _{ii}^0,...,\chi _{ii}^m,\chi _{i,1},...,\chi _{i,i-1})$ is always 1 for all $i=1,...,n$, i.e., it is a vector with a multinomial distribution. This means that if we have a set of parameters $\theta _r$, the expected value of the entries for all *i* in 1, 2, ..., *n* is given by

15 $$\begin{aligned} \mathbb {E}(\chi _{ii}^s|\theta _r)&=\hat{p}_{ii}^s=\frac{\mu ^s(x_i,y_i)}{\lambda _{\theta _0(t_i,x_i,y_i|H_{t_i}})} \mathbbm {1}(t_i\in S_s,E_s) \quad \text {with } s=0,1,...,m \end{aligned}$$

16 $$\begin{aligned} \mathbb {E}(\chi _{ij}|\theta _r)&=\hat{p}_{ij}=\frac{k(M_j)g(t_i-t_j)f(x_i-x_j,y_i-y_j|M_j)}{\lambda _{\theta _0(t_i,x_i,y_i|H_{t_i}})} \quad \text {with } j=1,...,i-1. \end{aligned}$$

The equations (15) and (16) correspond to the expectation step in the EM algorithm. The next step is to replace the $\chi$ values in (13) and (14) by their expected values. Using $\theta _r$ as the initial parameter value,

17 $$\begin{aligned} \ell _\text {I}(\theta _r)&= \sum _{s=0}^m \Big [ \sum _{i=1}^n \hat{p}_{ii}^s\log (\mu _{\theta _r}^s(x_i,y_i)\mathbbm {1}(t_i\in (S_s,E_s))\nonumber \\&\quad -\int _0^T \iint _S \mu _{\theta _r}^s(x_i,y_i)\mathbbm {1}(t\in (S_s,E_s)) dxdydt \Big ], \end{aligned}$$

18 $$\begin{aligned} \ell _\text {O}(\theta _r)&= \sum _{j=1}^n \Big [ \sum _{i>j} \hat{p}_{ij} \log \Big (k_{\theta _r}(M_j)g_{\theta _r}(t_i-t_j)f_{\theta _r}(x_i-x_j,y_i-y_j|M_j)\Big ) \nonumber \\&\quad - \int _{t_j}^T \iint _S k_{\theta _r}(M_j)g_{\theta _r}(t-t_j)f_{\theta _r}(x-x_j,y-y_j|M_j) dxdydt \Big ]. \end{aligned}$$

It is important to note that since the real branch structure (genealogy) of the earthquakes is unobservable, the equations (13) and (14) cannot be evaluated. However, when the real values are replaced by their expected values as in (17) and (18), they can be computed.

Another advantage of the EM approach is that all parameters concerning $\mu ^i$ are only in $(17)$, while the parameters $(A,\alpha ,c,p,d)$ are only in (18), therefore the optimization can be done separately. To solve (18) it is important to note that

$$\begin{aligned} \int _{t_j}^T \iint _S k_{\theta _r}(M_j)g_{\theta _r}(t-t_j)f_{\theta _r}(x-x_j,y-y_j|M_j) dxdydt \end{aligned}$$

can be rewritten as

$$\begin{aligned} k_{\theta _r}(M_j) \int _{t_j}^T g_{\theta _r}(t-t_j)dt \iint _S f_{\theta _r}(x-x_j,y-y_j|M_j) dxdy. \end{aligned}$$

The utility of taking the expressions in (4) and (5) is that the integrals can be solved easily, since

$$\begin{aligned} \int _{t_j}^T g_{\theta _r}(t-t_j)dt= 1-c^{p-1}(T-t_j+c)^{-p+1} \end{aligned}$$

and the spatial integral

$$\begin{aligned} \iint _S f_{\theta _r}(x-x_j,y-y_j|M_j) dxdy, \end{aligned}$$

which corresponds to the double integral of a bivariate Gaussian distribution, which can be solved efficiently using Monte Carlo methods. As an additional note,^53^ suggests the following approximation with different *k*, *g* and *f* functions

$$\begin{aligned} \int _{t_j}^T \iint _S k_{\theta _r}(M_j)g_{\theta _r}(t-t_j)f_{\theta _r}(x-x_j,y-y_j|M_j) dxdydt\approx k_{\theta _r}(M_j), \end{aligned}$$

this approximation holds if almost all aftershock activity occurs in the observed space and time, which is a very easy assumption to satisfy and it can lead to produce important reductions in computing time. We mention this approach because it has been used by different authors^32,38^, nevertheless, the implementation of algorithm 1 in this work does not use it because, as mentioned in^54^, it can generate inaccuracies in the estimation process.

Regarding (17), it is easy to find that the solution of

$$\begin{aligned}&\frac{{\partial }}{{\partial } \mu ^j_{uv}} \sum _{s=0}^m \Big [ \sum _{k=1}^n \hat{p}_{ii}^s\log (\mu _{\theta _r}^s(x_k,y_k)\mathbbm {1}(t_k\in (S_s,E_s))\\&\quad -\int _0^T \iint _S \mu _{\theta _r}^s(x_k,y_k)\mathbbm {1}(t\in (S_s,E_s)) dxdydt \Big ] =0 \end{aligned}$$

is

19 $$\begin{aligned} \hat{\mu }^j_{uv}=\frac{\sum _{k=1}^n \hat{p}^j_{ii} \mathbbm {1}((x_k,y_k)\in D_{uv}) \mathbbm {1}(t_k\in (S_j,E_j)) }{(E_j-S_j)\Delta x \Delta y}. \end{aligned}$$

The main advantage of using the piecewise expression of $\mu ^i$ is to retrieve a closed expression in (19), which enables a fast updating of the parameters. However, the main weakness of this approach is that only the earthquakes inside of $D_{kl}$ update the value of $\mu ^i_{kl}$, and a smooth estimation of $\mu ^i$ is not achieved.

To address the not well-conditioned problem we restrict the optimization problem, the parameter *A* must be less than 1, i.e. we are assuming that the smallest earthquake have less than one expected aftershock. Regarding the $\alpha$ value we followed the idea of Ross & Kolev^32^ of use the Helmstetter et al.^52^ inequalities.

Assuming the Gutenberg-Richter law^55^, the random variable ($M-M_0$) is distributed as an exponential random variable^56^ with rate $b\log (10)$, where *b* is the $b-$value of the Gutenberg-Richter law. Then, we can calculate the average number of offspring created per event, which is defined by

$$\begin{aligned} r:=\int _{M_0}^\infty Ae^{\alpha (M-M_0) } b\log (10)e^{-b\log (10)(M-m_0)}dM. \end{aligned}$$

In order to guarantee that *r* is finite and it is less than one, the following conditions must be fulfilled^52^

20 $$\begin{aligned} \alpha<b\log (10), \quad \frac{Ab\log (10)}{b\log (10)-\alpha }<1. \end{aligned}$$

Consequently, in this work we allow only parameters of *A* and $\alpha$ that satisfy the inequalities in (20).

During this study, we assumed a constant b-value over time and across the entire study region. This value was estimated using the maximum likelihood method^57^. Tests were also performed in Mexico using the b-positive estimator^42^ and the novel estimator presented by Lippiello & Petrillo^58^ called b-more-positive; however, the ETAS model estimators did not change significantly. Furthermore, Table 1 in Appendix App. 1 reports estimations assuming b-value lower and higher than those obtained via MLE, b-positive method and b-more-positive and the parameters did not show any important change with respect to those presented in “Mexico” section.

Although the choice of the MLE estimator or the family of b-positive estimators does not generate significant changes in our ETAS model estimators, we note that Nandan et al.^59^ introduced a variant of the ETAS model that allows different b-values to be considered. They speculate that the magnitude of the mainshock may affect the b-value for its triggered earthquakes. Also, Ito & Kaneko^60^, who, using continuum models of fully dynamic earthquake cycles with fault frictional heterogeneities, have observed that the b-value of foreshocks decreases with time prior to the mainshock.

Furthermore, as noted by^61^, the b-value also exhibits a spatiotemporal distribution, which we plan to analyze in detail in future work.

For *c* and *p* parameters, we restrict the acceptable values to be between [0, 5] and [1, 2]. We are being very flexible in our boundaries, since Holschneider et al.^62^ have observed that *c* and *p* are weakly identifiable.

In the case of *d*, with units deg$^2$, we restrict the optimization algorithm to be in (0,1), the idea of take 1 as the upper limit is be noninformative.

The maximization step in the EM algorithm consists in solving

21 $$\begin{aligned} \theta _{r+1}=\underset{\theta }{\operatorname {argmax}}\quad \ell _\text {I}(\theta _r) +\ell _\text {O}(\theta _r) \end{aligned}$$

Finally, by replacing *r* by ${r+1}$ the Expectation and Maximization steps must be iterated until $|\theta _r-\theta _{r+1}|<\varepsilon$, where $\epsilon >0$ is the convergence criterion. In this work, the optimization was done using the implementation of the *L-BFGS-B* algorithm in *python* using the implementation in *scipy*^63^.

As a summary of the EM algorithm that is used in “Results” section to detect the earthquake triggering effect of SSEs, the pseudo code is presented in the algorithm 1.

**Algorithm 1:**
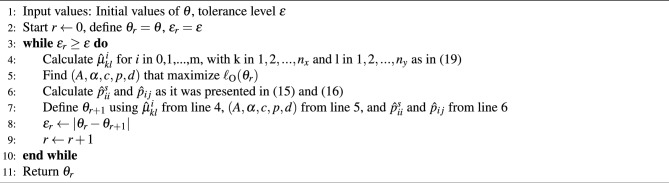
Expectation maximization algorithm of inhomogeneous spatio-temporal ETAS.

The initial values of $\hat{p}^s_{ii}$ and $\hat{p}_{ij}$ used in Algorithm 1 are defined by the following matrices

$$\begin{aligned} P^O&=\begin{pmatrix} 0 & 0 & 0& 0& \hdots & 0\\ \frac{1}{2} & 0 & 0 & 0& \hdots & 0\\ \frac{1}{3} & \frac{1}{3} & 0& 0& \hdots & 0\\ \frac{1}{4} & \frac{1}{4} & \frac{1}{4}& 0& \hdots & 0\\ \vdots & \vdots & \vdots & \vdots & \ddots & \vdots \\ \frac{1}{n} & \frac{1}{n} & \frac{1}{n} & \frac{1}{n} & \hdots & 0 \end{pmatrix}, \\ \quad P^I&= \begin{pmatrix} \frac{\mathbbm {1}(t_1 \in (S_0,E_0))}{\sum _{i=0}^m\mathbbm {1}(t_1 \in (S_i,E_i))} & \frac{\mathbbm {1}(t_1 \in (S_1,E_1))}{\sum _{i=0}^m\mathbbm {1}(t_1 \in (S_i,E_i))} & \hdots & \frac{\mathbbm {1}(t_1 \in (S_m,E_m))}{\sum _{i=0}^m\mathbbm {1}(t_1 \in (S_i,E_i))}\\ \frac{\mathbbm {1}(t_2 \in (S_0,E_0))}{2\sum _{i=0}^m\mathbbm {1}(t_2 \in (S_i,E_i))} & \frac{\mathbbm {1}(t_2 \in (S_2,E_2))}{2\sum _{i=0}^m\mathbbm {1}(t_1 \in (S_i,E_i))} & \hdots & \frac{\mathbbm {1}(t_2 \in (S_m,E_m))}{2\sum _{i=0}^m\mathbbm {1}(t_2 \in (S_i,E_i))}\\ \vdots & \vdots & \ddots & \vdots \\ \frac{\mathbbm {1}(t_n \in (S_0,E_0))}{n\sum _{i=0}^m\mathbbm {1}(t_n \in (S_i,E_i))} & \frac{\mathbbm {1}(t_n \in (S_2,E_2))}{n\sum _{i=0}^m\mathbbm {1}(t_n \in (S_i,E_i))} & \hdots & \frac{\mathbbm {1}(t_n \in (S_m,E_m))}{n\sum _{i=0}^m\mathbbm {1}(t_n \in (S_i,E_i))}, \end{pmatrix}, \end{aligned}$$

taking $\hat{p}^s_{ii}$ as the entry (*i*, *s*) of $P^I$ and $\hat{p}_{ij}$ as the entry (*i*, *j*) of $P^O$. The idea of the above matrices is to try to reflect the unknowns about the branching structure, starting with uniform distributions between being an aftershock or a background event, and uniformity among $\mu ^s$ that could generate the event if it is a background event.

All the codes used to fit the above model and generate the images in this work are available in https://github.com/isaiasmanuel/ETAS. The code used to perform Algorithm 1 is available in *EM2.py*. Also, the figures in this study were produced using the code *GNSSETAS_Figures.py*.

### Hypothesis testing

To verify if there is an improvement of our model, i.e., by adding $\mu ^i$ for $i = 1, 2,..., m$ with respect to the model which only has $\mu _0$, a hypothesis test must be performed to analyze the significance of the results. Due to the complexity of the model presented in Algorithm 1, it is not straightforward to derive the theoretical joint distribution of the estimators and determine the rejection regions. For this reason, we propose a Likelihood Ratio Test (LRT) based on parametric bootstrapping^64^.

Since bootstrap is a resampling approach that requires simulating synthetic earthquake catalogs, we will simulate the synthetic catalog in a similar way to Ross & Kolev^32^, based on the cluster process representation of the Hawkes process^65^, the advantage of this approach is avoiding the methodology of thining as in Fox et al.^38^ that requires more computational time. The simulation of synthetic catalogs is as follows

1. We simulate our synthetic mainshocks for each square $D_{uv}$ defined in the equation (12), and for each interval $i=1,2,...,m$, from a Poisson distribution with mean $\mu ^i_{uv}(E_i-S_i)\Delta x\Delta y$, the occurrence time of this event is simulated from a uniform distribution in $(S_i,E_i)$ and the magnitude is simulated from the Gutenberg-Richter law with the $b-$value estimated via maximum likelihood from the real catalog^57^.
2. For each simulated earthquake *j* that it is direct offspring have not been calculated previously, we simulate the number of direct offspring from a Poisson distribution with mean $Ke^{\alpha (M_j-M_0)}$, and the offspring locations and times are simulated directly from random variables with densities given by equations (4) using $t=t_j$ and (5) using $(x_j,y_j)$. If the new earthquakes have times larger than $E_0$ or if they are outside of our study area, they are discarded.
3. Repeat the previous step until no new offspring is generated.

The code to simulate synthetic earthquake catalogs is available in *Hypothesis_testing.py*.

The hypothesis to test in the LRT are

- $H_0$: $\theta$ has $\mu ^i$ for $i=1,2,..$ equal to 0
- $H_1$: $\theta$ has at least one $\mu ^i$ for $i=1,2,..$ different to 0

From the real catalog $\{x_i,y_i,t_i,m_i\}_{i=1}^n$ we can obtain the maximum likelihood estimators (MLE) of $\theta$ using the complete model and the model that only has $\mu ^0$, which will be denoted by $\hat{\theta }$ and $\tilde{\theta }$ respectively, then our test statistic is defined by

$$\begin{aligned} T_{LR}=\frac{L_1(\hat{\theta })}{L_0(\tilde{\theta })}, \end{aligned}$$

where $L_1$ and $L_0$ denote the likelihoods of the complete and reduced model. In order to define the rejection region for our hypothesis testing, we will generate *B* synthetic catalogs $\{x_i,y_i,t_i,m_i\}_{i=1}^{n_b}$, with $b=1,...,B$, using $\tilde{\theta }$ from the reduced model. Once the synthetic catalogs are obtained, we estimate the MLE for both models to each synthetic catalog to obtain a pair $\hat{\theta }_b$ and $\tilde{\theta }_b$, which allows to define

$$\begin{aligned} T_{LR,b}= \frac{L_{1,b}(\hat{\theta }_b)}{L_{0,b}(\tilde{\theta }_b)}, \end{aligned}$$

where $L_{0,b},L_{1,b}$ denote the Likelihood functions of the reduced and complete model using the synthetic catalogue *b*. It is important to note that the idea of the bootstrap methodology is to obtain an approximation of the distribution of our estimators through empirical distributions obtained by resampling^64^.

Finally we will reject $H_0$ in favor of $H_1$ at a confidence level $\gamma$ if the following expression is fulfilled

$$\begin{aligned} \frac{\#\{T_{LR,b}:T_{LR,b}>T_{LR}\}}{B}\le \gamma , \end{aligned}$$

where $\#$ denotes the cardinality of the set.

While the LRT allows us to determine whether there is evidence that the full model is preferable to the reduced model, it is not informative in terms of seeing which $\mu ^i_ {uv}$ are contributing to that conclusion. Taking this into account and the fact that we already calculate $\hat{\theta }_b$ for all synthetic catalogs, we can examine marginally which $\mu ^i_{uv}$ from $\hat{\theta }$, denoted by $\hat{\mu }^i_{uv}$, are significantly greater than the corresponding $\mu ^i_{uv}$ from $\hat{\theta }_b$, denoted by $\mu ^{i,b}_{uv}$. i.e., we can define

$$\begin{aligned} T_{uv}=\frac{\#\{\mu ^{i,b}_{uv}:\mu ^{i,b}_{uv}\ge \hat{\mu }^i_{uv}\}}{B}, \end{aligned}$$

and conclude that $\hat{\mu }^i_{uv}$ is significantly higher than $\mu ^i_{uv}$ under the reduced model at level $\gamma$ if $T_{uv}\le \gamma$.

Note that the value $\mu _{uv}^i$ for $i=1,2,...$ can be 0 if no earthquake was observed in the square $D_{uv}$ during the interval $(S_i,E_i)$ or if the observed earthquakes were classified as offspring of an earthquake that ocurred prior to $(S_i,E_i)$.

In the “Results” section this hypothesis testing methodology is applied for records in Guerrero, Mexico and the Boso Peninsula, Japan.

Additionally, since we are obtaining synthetic catalogs with a sample size $n_b$, we select the size of our mesh in equation 12 as the $n_x=n_y$ that minimize

$$\begin{aligned} \frac{1}{B} \sum _{b=1}^B (n-n_b)^2. \end{aligned}$$

In other words, the mesh size is selected through minimize the mean squared error of the number of earthquakes. It is worthy to mention that Asim et al.^66^ have proposed the idea of use a multiresolution mesh to improve the computational time, nevertheless due the size of our region a non multiresolution mesh is still computationally affordable.

## Data

### Mexico

In Fig. 1, all the epicenters in the study are shown in blue, on a mesh with $n_x=n_y=14$, following the notation in equation (12). The earthquake catalog was obtained from the Advanced National Seismic System (ANSS) ^67^, with dates from 2000/01/01 to 2016/12/31, and magnitudes greater than or equal to 4.3. Only the earthquakes related to plate subduction (i.e., on the landward side of the trench) were considered in this study. The magnitude completeness of the ANSS catalog in the study region was assessed using the Maximum Curvature Method^68^, and the magnitude of completeness (Mc) was found to be M4.0. However, as this method is known to systematically underestimate Mc by a fraction of a unit^69^, M4.3 was used as the threshold $M_0$ in equations (3) and (5) in the present work.

On September 8, 2017, an earthquake of M8.2 occurred in Mexico and some studies, such as^70^, have suggested that it may have broken the entire subducted Cocos lithosphere and significantly altered the seismicity in the subduction system. For this reason, we limit our study period to the end of 2016.

**Fig. 2:**
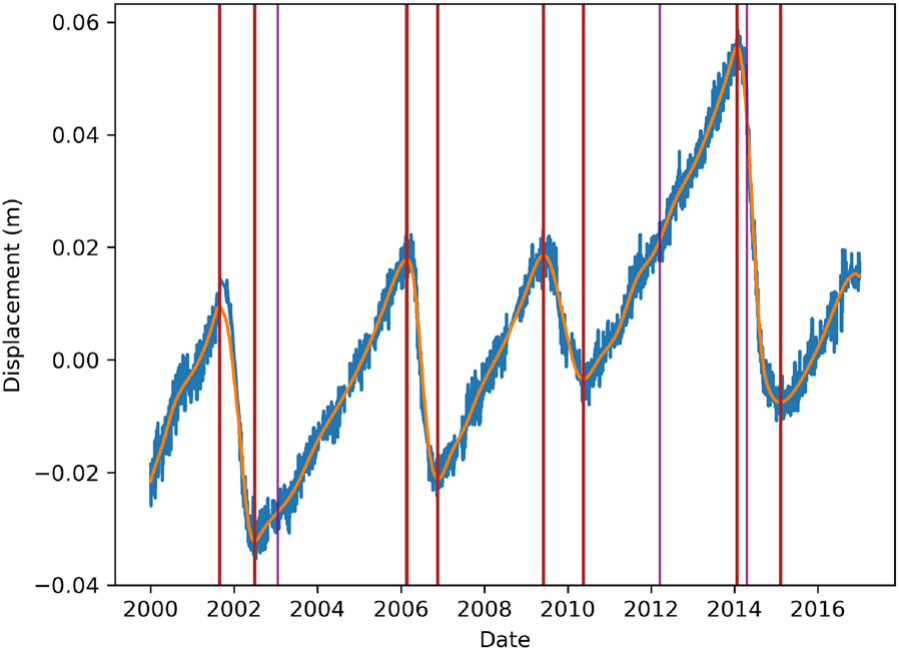
Global navigation satellite system data at Cayaco station. North-to-south component at Cayaco station (blue). Fitted b-spline polynomial (orange). The periods when the data slope is negative is indicated by vertical red lines, and the purple vertical lines are when an earthquake with $M>7$ ocurred.

In Fig. 2, the North-South component of the GNSS time series of the Cayaco (CAYA) continuous GPS monitoring station is presented. CAYA is located in Guerrero state, Mexico, at $-100.2672\hbox {E}^{\circ },17.0485\hbox {N}^{\circ }$. The duration of SSEs can be defined as the periods of southward displacements at this station^9^, and the SSEs appear to have a periodicity of approximately four years. This GNSS data is available in https://github.com/isaiasmanuel/ETAS as *caya_ns*.

To estimate the start and end times ($S_i$ and $E_i$) of the SSEs, b-splines^75^ are fitted to the raw data. The duration of the SSEs is defined by the periods when the spline has a negative slope, as shown in Fig. 2. The data for this work was provided by the Servicio Sismológico Nacional (SSN) of Mexico and by the Department of Seismology, IGEF UNAM.

The SSE slip contour curves with 4 cm of slip spacing (from 2 cm to 18 cm) of the first 3 SSEs (the 2001-2002, 2006, and 2009-2010 SSEs) have been digitalized from Radiguet et al.^73^. The 15 cm slip contour of the 2014 SSE was digitalized from^4^ and is presented in Figure 1. The expected result of the algorithm  1 is that if an earthquake triggering effect of SSEs exists, $\mu ^i$ with $i=1,2,3,4$ should have an increase near the SSE slip contours.

### Japan

To test the flexibility and capability of our model, we additionally explored the Boso Peninsula, located in the Sagami Trough subduction zone in Japan. The seismicity-triggering effect of SSEs and swarm activity produced during SSE periods in the Boso Peninsula have been previously studied by Okutani & Ide^22^ and Fukuda^30^. For this analysis, we used data from the Japanese Meteorological Data^76^ from 2001/01/01 to 2009/01/01 and $M_0=3$. Accoding to the geodetic analysis by Fukuda^30^, during this period, two SSE ocurred from 2002/10/01 to 2002/10/19 and from 2007/08/12 to 2008/08/25. These periods were used to define $\mu ^1$ and $\mu ^2$, respectively.

**Fig. 3:**
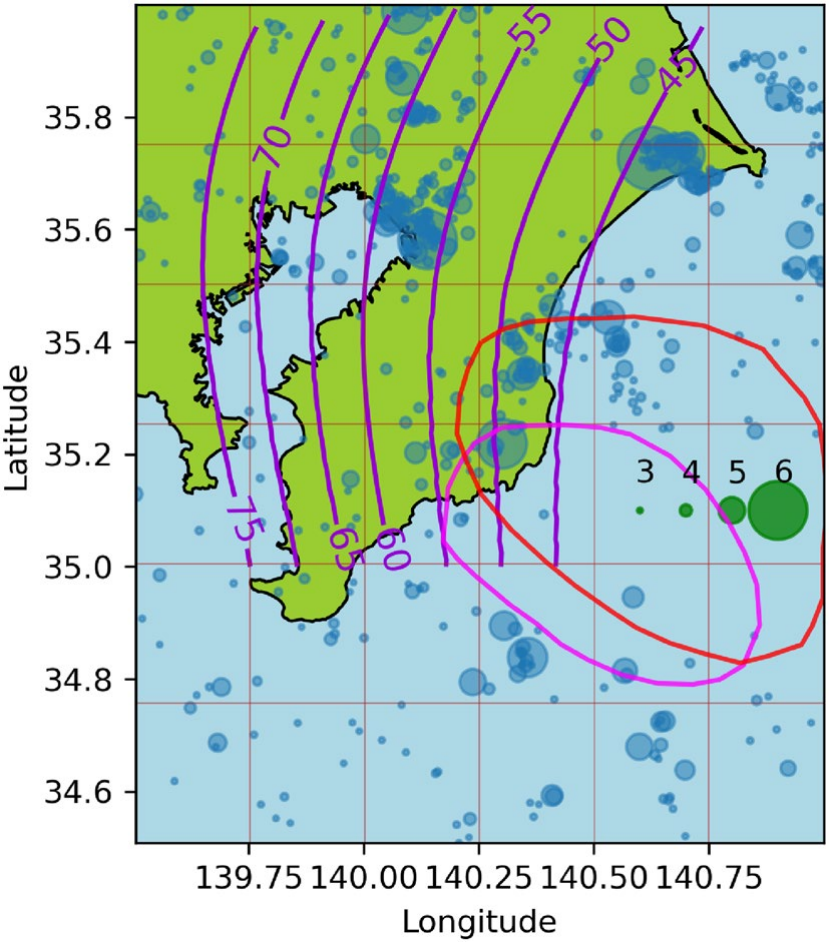
Seismicity data from the Boso Peninsula, Japan. The colors are the same as in Fig. 1, except for the SSEs. The fuchsia and red contours denote the daily slip rate distributions of $4\frac{\text {m}}{\text {year}}$ for the 2002 and 2007 SSEs, respectively, digitized from^30^.

Figure 3 shows the seismicity during our study period, and the mesh used in Algorithm 1, in this example $n_x=n_y=6$. The daily slip rate distributions of $4\frac{\text {m}}{\text {year}}$ for the 2002 and 2007 SSEs are also presented. The digitized data correspond to the slip rates on 2022/10/08 and 2007/08/16 from^30^, which represent the days with the highest slip rates.

It is important to note that, while the SSEs in Mexico occurred over several months, those in the Boso Peninsula lasted only several weeks. This substantial difference in time scale is a key reason for analyzing the performance of our model in both regions.

## Results

### Mexico

**Fig. 4:**
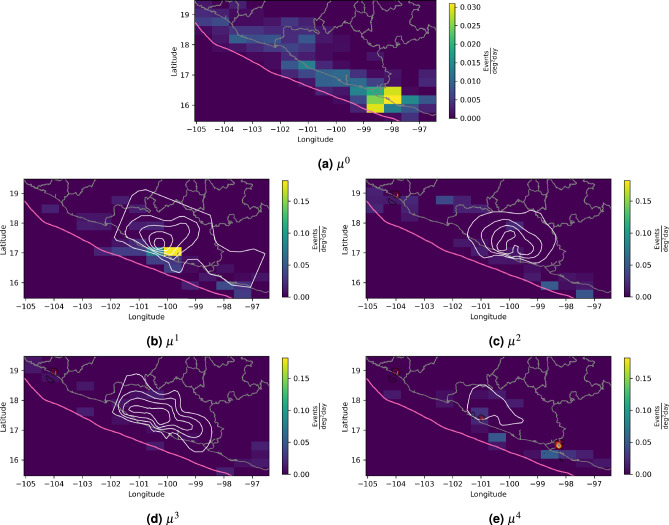
Estimated background seismicity for the Mexican dataset. $\mu ^i$ with i=0,1,2,3,4 are estimated using Algorithm 1. The white contours in $\mu ^1$, $\mu ^2$ and $\mu ^3$ represent the slip distributions with 4-cm slip intervals for the 2001-2002, 2006, and 2009-2010 SSEs, respectively, digitized from^73^. In $\mu ^4$, the slip contour of the 2014 SSE is shown.

**Fig. 5:**
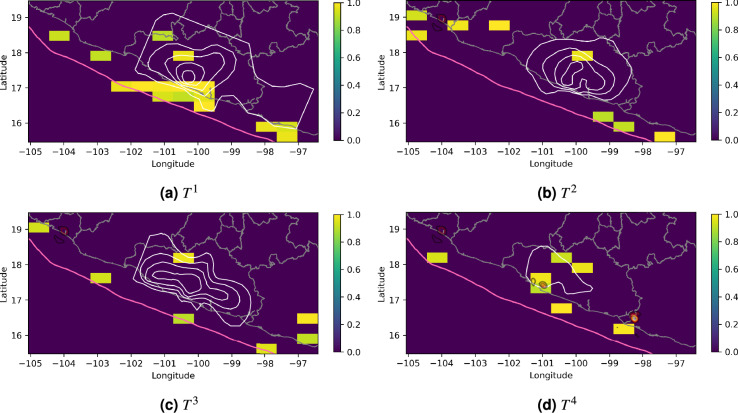
Significant $T^i$ values for the Mexican dataset. The $T^i$ values with $i=1,2,3,4$ that are significantly different from zero are shown.

Using $\mu ^1$, we observe a substantial increase in the background seismicity of 0.18$\frac{\text {Events}}{\text {deg}^2 \text {day}}$, compared to the stationary background rate of 0.01 given by $\mu ^0$, during the 2001-2002 SSE in the square with a vertex at $-100.11\hbox {E}^{\circ }$, $17.18\hbox {N}^{\circ }$ (Fig. 4). It is important to note that this area is located close to the regions with the largest slip gradient in the slip contours of Fig. 4.The significant influence of $\mu ^1$ on the background seismicity is further supported by Fig. 5, which highlights the squares with a significant $T_{uv}^i$ at the $90\%$ confidence level using $B=100$. Additionally, the LRT rejects the null hypothesis (p-value = 0.09) in favor of our model.

Since SSE slips release stored elastic energy, the regions with the largest SSE slips have experienced a reduction in cumulative stress. In contrast, stress increases just outside the margins of the SSE patches, where an increase in background seismicity can be expected due to the triggering effects. This is consistent with our estimation in Fig. 4.

Regarding $\mu ^2$ and $\mu ^3$, we observe values greater than zero next to the epicenter of the 2003/01/22 earthquake and in the margins of the 2006 and 2009-2010 SSEs. Nevertheless, these values are too small and dispersed to conclude that a triggering effect could be visible. Furthermore, as shown in Fig. 5, the observed values in nearly all the squares are not statistically significant.

Radiguet et al.^4^ have discussed that the 2014/04/18 M7.3 Papanoa earthquake was triggered by the 2014 SSE. Consistently, our model shows a significant increase in the background seismicity rate during the SSE period in the square that contains the epicenter, as well as in the square with vertex at $-100.73\hbox {E}^{\circ }$, $16.89\hbox {N}^{\circ }$ where, according to^4^, the SSE caused a slip of approximately 0.05 m.

We also found a significant value of $\mu ^4$ in squares with vertices at $-98.88\hbox {E}^{\circ }$, $16.03\hbox {N}^{\circ }$ and $-98.26\hbox {E}^{\circ }$, $16.89\hbox {N}^{\circ }$ which are next to the epicenter of the 2012/03/20 earthquake. Although we have focused this work on discussing changes in seismicity during periods of SSEs, it is important in future work to consider the background seismicity variations around large earthquakes to account for possible tectonic stress rearrangements.

While $\mu ^1$ reached the highest values among all $\mu ^i$, $\mu ^2$ and $\mu ^3$ exhibited much lower levels of activity. This may be related to differences in the source characteristics of each SSE (i.e., moment magnitude, duration, and slip distribution).

In terms of moment magnitude, the 2001–2002 SSE was the largest (Mw 7.65), while the 2006, 2009–2010, and 2014 SSEs had smaller magnitudes of Mw 7.49, Mw 7.54, and Mw 7.60, respectively, according to Radiguet et al.^4,73^. The duration of the 2001–2002 SSE was 436 days, while the other three SSEs lasted 314, 510, and 378 days, respectively.

In particular, the 2009-2010 SSE exhibited relatively small moment magnitude and longest duration, indicating a lower moment rate. This may explain our observation that this SSE did not produce a detectable triggering effect for earthquakes of M4.3 or larger.

**Fig. 6:**
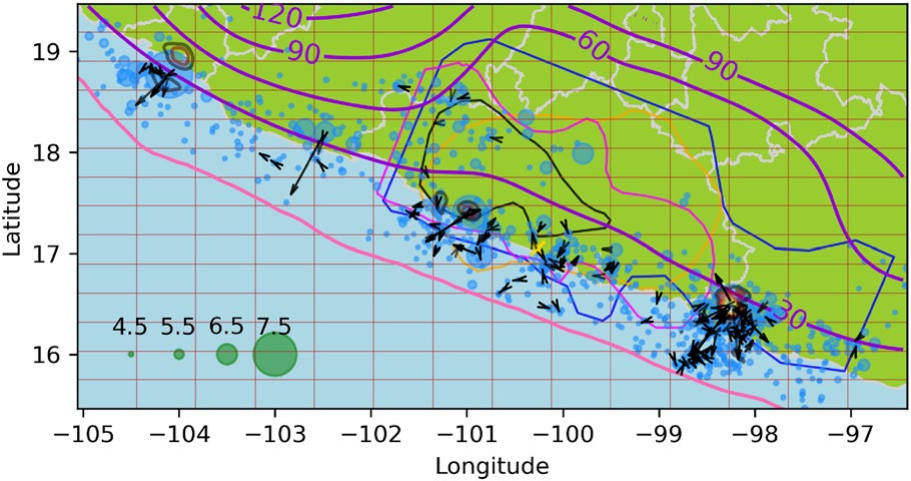
Offspring estimation for the Mexican dataset. Map of earthquakes with arrows pointing from events to their offspring according to $P^O$.

To show the validity of the estimation presented in this work, the genealogy of earthquakes defined by the estimators obtained using Algorithm 1, is presented in Fig. 6. In this figure, all the earthquakes in our dataset are shown with arrows pointing to their aftershocks, in the figure only arrows where $P^O_{ij}\ge 0.5$ are presented.

An anomalous arrow was observed originating from the earthquake with coordinates $-102.540\hbox {E}^{\circ }$, $18.026\hbox {N}^{\circ }$. This may be due to the fact that the time between both earthquakes was only 6.86 hours, and the fact the second earthquake is in a square where only 3 events were recorded throughout the entire study period. This arrow may be an unreliable result.

**Fig. 7:**
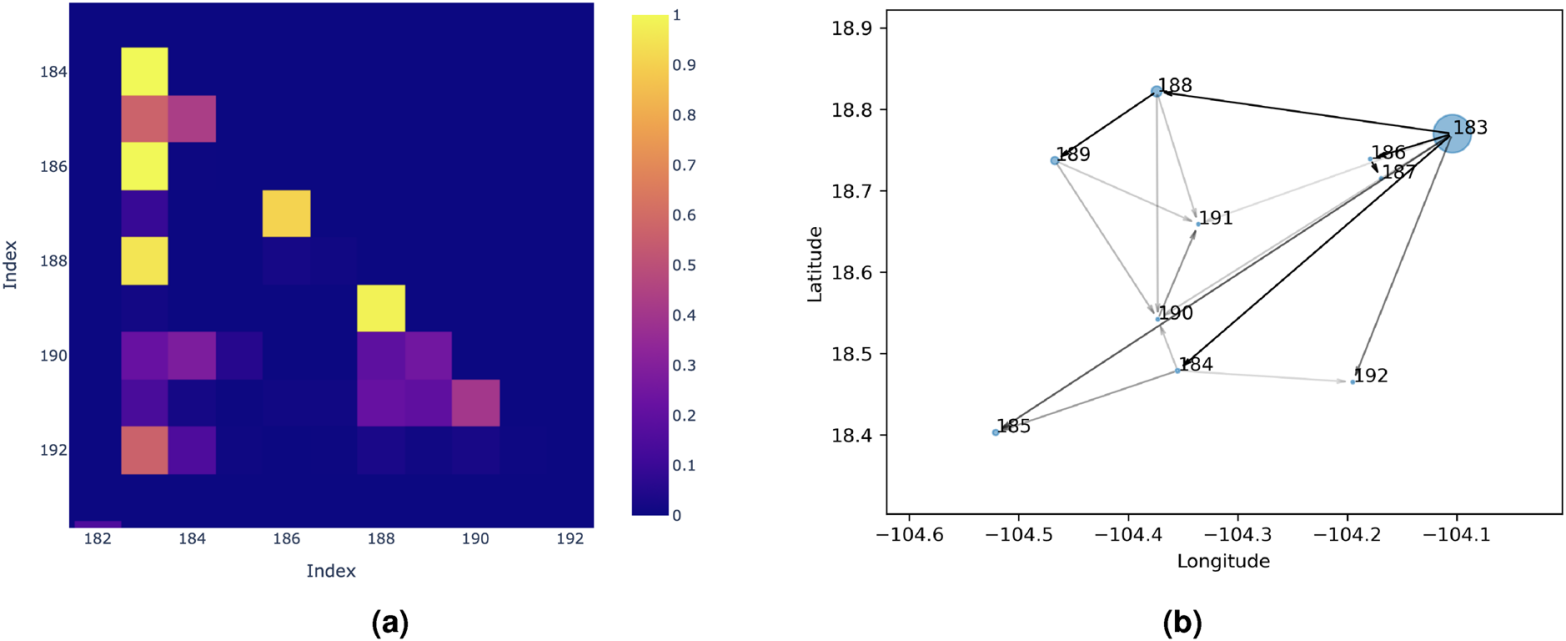
Genealogy of events 183-192. In (**a**), the matrix $P^O$ for indices 183 to 192 is shown.  (**b**) Presents the same events as a genealogy.

To clarify the results in Fig. 6, a zoomed-in view of indices 183 to 192 is presented in the Fig. 7a. This window was selected because it contains the largest earthquake (event 183) in our data set, with a magnitude of 7.6. As it can be seen in the Fig. 7a, earthquake 190, with an epicenter at -104.37E$^\circ$,18.54N$^\circ$, could be an offspring of the earthquakes 183,184, 188 or 189. Therefore, the 4 arrows pointing to it are lighter in color than the arrow between earthquakes 183 and 184, which have an associated probability close to 1.

Figure in 7b allows not only to see which earthquakes are background events and which are offsprings, but also to recover an estimated entire genealogy—an idea that has been previously explored in the works of Rasmussen^48^ and Fox et al.^38^.

**Fig. 8:**
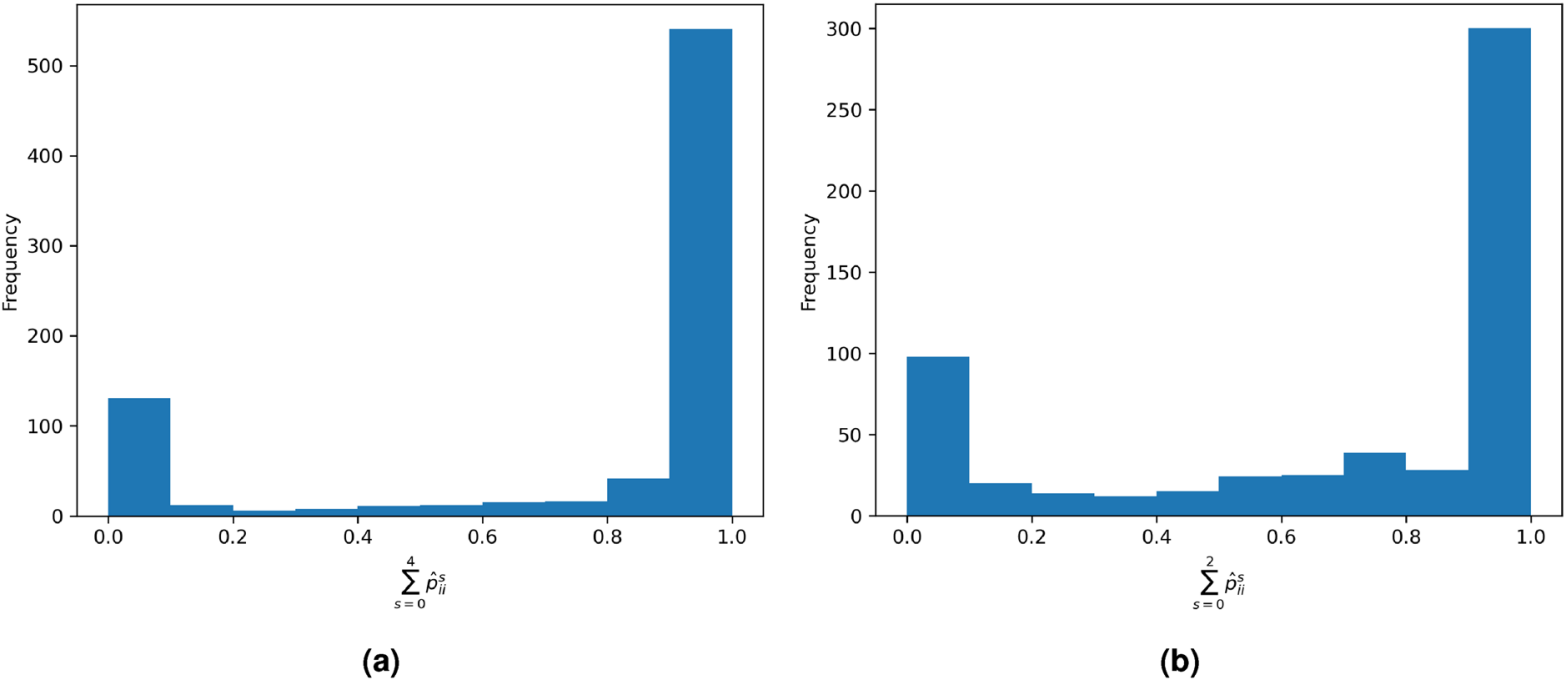
Background probability histogram. Histograms of probabilities of being a background event for all earthquakes in our dataset are presented. (**a**) Mexican dataset (**b**) Japanese dataset.

The b-value for our dataset is 1.254 and the estimated vector of aftershock parameters $(A,\alpha ,c,p,d)$ is (0.118 events, 1.112, 0.021 days, 1.363, 0.0048 deg$^2$). The expected total value of earthquakes using the data in this section and Algorithm 1 is

$$\begin{aligned} \int _{0}^T \iint _S \lambda (t,x,y|\mathcal {H}_t)=774.15, \end{aligned}$$

using our sample of 794 earthquakes. Figure 8a shows a histogram of the values (i.e., the probability of each earthquake being a background event):

$$\begin{aligned} (1-\rho _j)=\sum _{s=0}^4 \hat{p}_{ii}^s. \end{aligned}$$

In this figure, we observe a bimodal density concentration near 0 and 1, which is similar to the one presented by Zhuang et al.^15^. The probability of being an aftershock is considerably lower. This could be due to the use of $M_0=4.3$, because, as shown in Fig. 8b, for the Boso Peninsula in Japan ($M_0=3$), there are more aftershocks in proportion than in the Mexican case.

### Japan

**Fig. 9:**
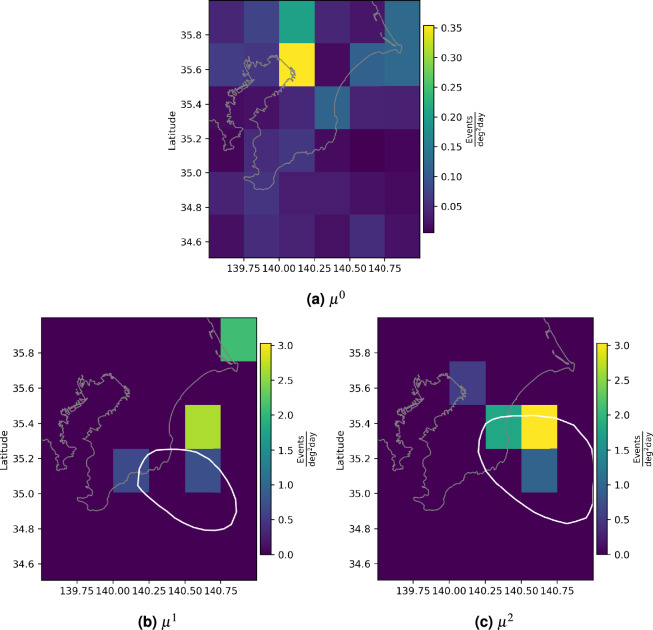
Estimated background seismicity for the Japanese dataset. $\mu ^i$ with i=0,1,2 estimated using Algorithm 1 are shown. The white contours represent the daily slip rates presented in Fig. 3 for the 2002 and 2007 SSE.

According to^30^, the magnitudes of the observed SSEs are 6.67 and 6.65 for the 2002 and 2007 events, respectively. Although they had shorter durations than the SSEs in Guerrero, Mexico, the SSEs in the Boso Peninsula, Japan, have exhibited notable increases of the background seismicity rate, as it can be observed in Fig. 9. In both cases, the square with the highest value has a vertex $140.5\hbox {E}^{\circ }$, $35.75\hbox {N}^{\circ }$, where $\mu ^1$ and $\mu ^2$ reach values of 2.65 and 3.02, respectively, while $\mu ^0$ for the same square was 0.03.

**Fig. 10:**
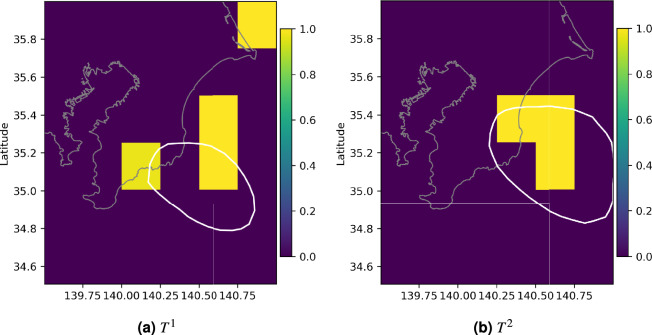
Significant $T^i$ values for the Japanese dataset. The $T^i$ with $i=1,2$ that are significantly different from zero are presented.

For both SSEs, $T^i$ for the squares associated with the maximum value of $\mu ^1$ and $\mu ^2$ was statistically significant, as shown in Fig. 10, and the the LRT rejects the null hypothesis (p-value = 0.01) in favor of our model. Also, as expected, the increase in the background seismicity rate was observed in the margins of the SSE patches. This is consistent with the Mexican results

**Fig. 11:**
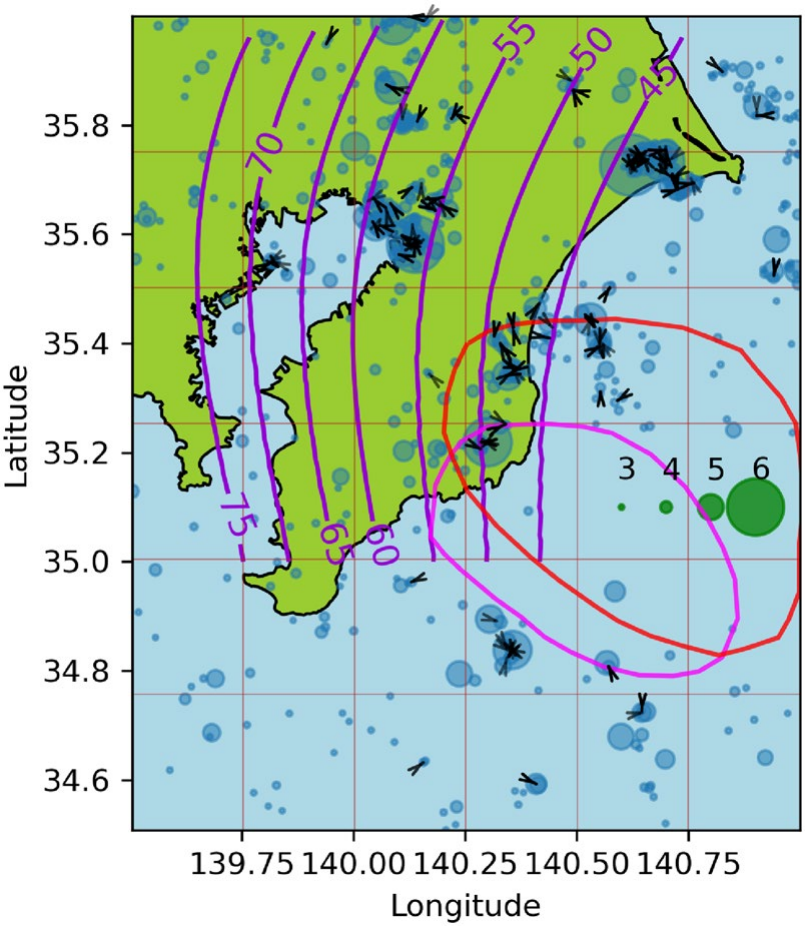
Offspring estimation for the Japanese dataset. Map of earthquakes with arrows pointing from events to their offspring according to $P^0$.

**Fig. 12:**
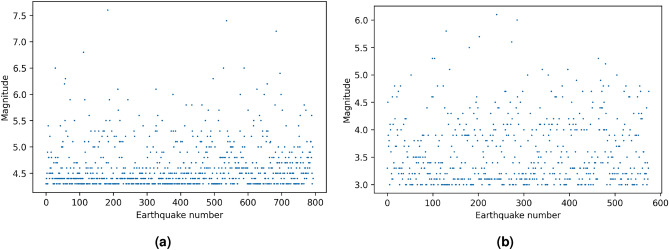
Ranked time vs magnitude. (**a**) Mexican dataset (**b**) Japanese dataset.

**Fig. 14:**
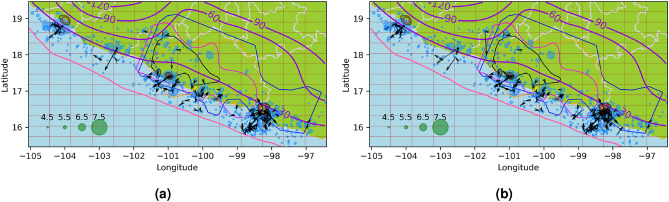
Mexican genealogy using alternative kernels. (**a**) Bivariate Gaussian (**b**) Bivariate t kernel.

The b-value for this data is 0.709, and the estimated vector of aftershock parameters $(A,\alpha ,c,p,d)$ is (0.365 events, 1.03, 0.002 days, 1.029, 4.101e-05 deg$^2$). And we obtain

$$\begin{aligned} \int _{0}^T \iint _S \lambda (t,x,y|\mathcal {H}_t)=554.10, \end{aligned}$$

for our sample of 575 earthquakes. As in “Mexico” section, Fig. 11 presents the estimated genealogy.

## Discussion and conclusions

We have successfully developed an inhomogeneous spatio-temporal ETAS model. Our approach extends the ideas of Fox et al.^38^ by introducing a piecewise constant spatial background seismicity over time, which contrasts with their non-time-dependent approach. Furthermore, this approach can be regarded as the spatio-temporal extension of the model proposed by Okutani & Ide^22^, which considers only the temporal components.

We also extended the work of Li et al.^16^ by employing a marked point process. Furthermore, we did not assume that spatial and time contributions of the background seismicity interact solely through multiplication, and we accounted for differences in the spatial distribution of increased background seismicity for each SSE.

Unlike Marsan et al.^27^ and Reverso et al.^28^, which rely on subjectively chosen local spatio-temporal windows, we used the estimation of the SSE durations from GNSS data and utilized them in our seismicity modeling. Moreover, rather than using an approximate approach that sequentially optimizes changes in the background seismicity rate across numerous small spatio-temporal windows, our method simultaneously performs a maximum likelihood estimation of spatio-temporal variations in the background seismicity rate over the entire analysis region and period, which represents a key technical advance of our model.

Furthermore, for the first time, we quantitatively evaluated the relationship between seismic activity and SSEs in the Mexican subduction zone using a statistical seismicity model that accounts for not only regular earthquakes but also SSEs. Our results from Mexico demonstrate statistically significant increases in the background seismicity accompanying Guerrero SSEs, highlighting the seismological novelty of this study.

Our model also successfully detected statistically significant increases in background seismicity associated with SSEs in the offshore Boso Peninsula region of Japan. Despite the substantial differences in the time scales of SSEs between the Boso Peninsula and Guerrero, Mexico, consistent results were obtained—namely, an increase in background seismicity near the margins of the SSE patches. This demonstrates the adaptability and broad applicability of our model to different datasets.

The assumptions made for our model were flexible enough to be applied in different regions, and we expect to continue improving the model and exploring other plate boundaries where SSEs occur, such as in New Zealand and Peru^12,13^. Applying our model to these regions will enable a deeper understanding of how SSEs trigger fast earthquakes.

In future work, we plan to extend our algorithm to allow dependencies between mainshock and aftershock magnitudes. Currently, we model magnitudes independently. However, it is important to explore possible dependencies between magnitudes, as Nandan et al.^59^ found evidence of such dependencies in California, USA.

We also intend to improve our algorithm by replacing the piecewise estimators of $\mu ^i$ with smooth functions in the space domain. In the context of the ETAS model, interesting ideas have recently been explored for this purpose, such as the use of Dirichlet processes^32^ (DP), or Gaussian processes^35^ (GP). An additional advantage of using DP or GP is that they allow inference to be performed within a Bayesian framework.

## Figures and Tables

**Fig. 13:**
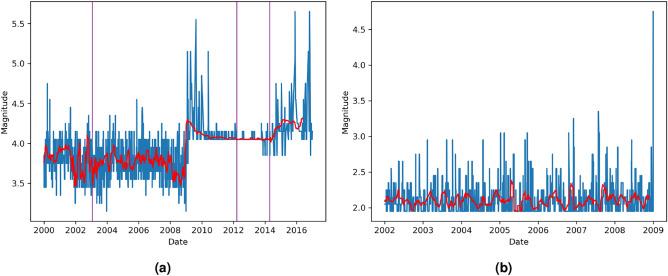
Maximum curvature magnitudes. (**a**) Mexican dataset (**b**) Japanese dataset. In purple the date of earthquake with magnitude greater than 7, and in red a moving average taking 50 points.

**Fig. 15:**
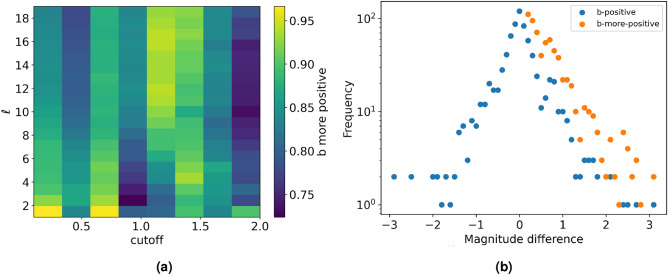
b-more-positive estimator for the Mexican dataset. (**a**) b-more-positive estimator for different cutoffs and lags, (**b**) Magnitude differences used for each estimator, with $\ell =10$.

## Data Availability

Data is provided within the manuscript.

## Appendix 1: Additional figures

As mentioned in Section 2.1, if a catalog exhibits STAI after strong earthquakes, a reduction in the occurrence of small earthquakes would be expected, which is not evident in Fig. 12. We also estimate the maximum curvature magnitude using 10-day bins for our catalog, as shown in Fig. 13. For the Mexican case, we set the cutoff magnitude at 4.3, primarily to reduce the bias introduced by the change in the mean in 2009, while for the Japanese case, we set it at 3 to reduce the computational load. We also do not see any substantial changes associated with large earthquakes in this figure (Fig. 14).

**Table 1:** Parameter values for different *b*-values.

*b*-value	*A*	$\alpha$	*c*	*p*	*d*
0.6	1.178e$-01$	1.121e$+00$	2.121e$-02$	1.358e$+00$	4.724e$-03$
2.5	1.180e$-01$	1.106e$+00$	2.173e$-02$	1.372e$+00$	4.979e$-03$

With the aim of obtaining more robust b-value estimators, van der Elst^42^ proposed the b-positive estimator. Instead of work with a sample of magnitudes $m_i$, $i=1,2,...,m$, which are assumed to follow the Guttenberg Richter law^55^, that is, independent exponential random variables with rate $b\log (10)$ shifted by $M_c$, he proposed using the auxiliary variables $Z_i=|m_{i+1}-m_i|$ with $i=1,2,...,m-1$. He defines the b-positive estimator as

$$\begin{aligned} b_+(M^\prime _c)=\Big ( \frac{\sum _{i=1}^{m-1} Z_i\mathbbm {1}(Z_i\ge M^\prime c)}{\sum _{i=1}^{m-1} \mathbbm {1}(Z_i\ge M^\prime c)} - M^\prime c\Big )^{-1}\log (e). \end{aligned}$$

In his work, he exhibited through simulations that the estimator can improve the estimation of the b-value, particularly in the presence of incompleteness as modeled by Ogata & Katsura model^77^.

One of the main concerns regarding the b-positive estimator is that if we only use the $Z_i$ where $\mathbbm {1}(Z_i\ge M^\prime c)$, the sample size decreases significantly, in order to solve this problem, Lippiello and Petrillo^58^ have proposed the b-more-positive estimator, they added an extra hyperparameter *l* to define

$$\begin{aligned} \vartheta _i^l=\min \{i: |m_{i+j+1}-m_{i+j}|>M^\prime c, j=0,..,{l-1}\}, \end{aligned}$$

for the non empty sets, which it allows to define

$$\begin{aligned} Z_i^\prime =|m_{i+\vartheta _i^j+1}-m_{i+\vartheta _i^j}|. \end{aligned}$$

Then the b-more-positive estimator is defined as

$$\begin{aligned} b_{+l}(M^\prime _c)=\Big (\frac{1}{|\mathcal {I}|}\sum _{\mathcal {I}}\ Z_i - M^\prime c\Big )^{-1}\log (e). \end{aligned}$$

where $\mathcal {I}$ is the set of indexes which $\{i: |m_{i+j+1}-m_{i+j}|>M^\prime c, j=0,..,{l-1}\}$ is non empty. As can be seen in Fig. 15b, this allows an increase in the sample size for our estimation, nevertheless, it is important to note that this increases the dependence between the $Z_i^\prime$ variables compared to the $Z_i$ in the b-positive estimator.

Lippiello & Petrillo studied the performance of the b-more-positive estimator through numerical simulations, in their example the b-more-positive showed an improvement with respect to the b-positive approach, in part because the b-positive estimator is a particular case of the b-more-positive one if we take $l=0$.

Although estimators have proven useful, new areas of research on this new type of estimator emerges, mainly due to the lack of independence of the $Z_i^\prime$ variables and how to obtain maximum likelihood estimators since the current b-positive and b-more-positive estimators rely on the pseudolikelihood. Although this facilitates the calculation of estimators, it remains an approximation of the likelihood.
